# Supplementary material for: Haplotype Analysis of BADH1 by Next-Generation Sequencing Reveals Association with Salt Tolerance in Rice during Domestication
Source: Int J Mol Sci. 2021 Jul 15;22(14):7578. doi: 10.3390/ijms22147578 (PMC8305476; doi:10.3390/ijms22147578)
Supplement: Supplementary file 1 [file ijms-22-07578-s001.zip › Captions of Supplementary Materials Figures and Tables.pdf]

Supplementary Materials

Haplotype Analysis of *BADH1* by Next-Generation Sequencing Reveals Association with Salt Tolerance in Rice during Domestication

Myeong-Hyeon Min [1,†], Thant Zin Maung [1,†], Yuan Cao [1], Rungnapa Phitaktansakul [1], Gang-Seob Lee [2], Sang-Ho Chu [3], Kyu-Won Kim [2] and Yong-Jin Park [1,3,*]

[1] Department of Plant Resources, College of Industrial Science, Kongju National University, Yesan 32439, Korea; mmh7272@gmail.com (M.-H.M.); tzmaung.yau2009@gmail.com (T.Z.M.); yuancao2017@gmail.com (Y.C.); phitaktansakul2017@gmail.com (R.P.)
[2] Agricultural Biotechnology Department, National Institute of Agricultural Sciences, Rural Development Administration, Jeonju 54874, Korea; kangslee@korea.kr (G.-S.L.); kyuwonkim@kongju.ac.kr (K.-W.K.)
[3] Center of Crop Breeding on Omics and Artificial Intelligence, Kongju National University, Yesan 32439, Korea; sanghochu76@gmail.com
* Correspondence: yjpark@kongju.ac.kr; Tel.: +82-41-330-1201; Fax: +82-41-330-12
† These authors contributed equally to this work.

Supplementary Materials: The following are available online at www.mdpi.com/xxx/s1.

Supplementary Table S1: Passport information for 475 rice accessions

Supplementary Table S2: A total number of genetic variations of *BADH1* in 421 cultivated rice and 54 wild rice accessions. The cultivated rice was subdivided into subpopulations (ecotypes), Temperate Japonica, Tropical Japonica, Indica, Aus, Aromatic, and Admixture. The number of variants was counted under each ecotype of cultivated rice, but in wild rice, the number were counted under each species (21 species). SNP: single nucleotide polymorphism

Supplementary Table S3A: Nucleotide diversity (π-values) of *BADH1* in 421 cultivated rice accessions. The diversity values were patterned in 10,000 bp (10 kb) sliding window size and taken out for different classified rice groups, Temperate Japonica, Tropical Japonica, Indica, Aus, Aromatic, and Admixture, including the whole rice accession.

Supplementary Table S3B: Tajima's D values of *BADH1* in 421 cultivated rice accessions. The Tajima's D values were patterned in 10,000 bp (10 kb) sliding window size and taken out for different classified rice groups, Temperate Japonica, Tropical Japonica, Indica, Aus, Aromatic, and Admixture, including the whole rice accession.

Supplementary Table S4: Haplotyping revealed the total number all SNPs (single nucleotide polymorphism) and InDels (insertions and deletions) detected in the gene region of *BADH1* in 421 rice accessions. Based on their similar genetic variations, there were 39 haplotype groups (here, we used as 'Hap', indicating their functional SNPs in both exons and introns. There were in total 224 alleles (146 SNPs and 78 InDels) detected in the whole *BADH1* gene region. Condon changes together with amino acid transition due to functional SNP substitutions were also supported at each position of the coding regions.

Supplementary Table S5A: List of haplogroups for selected haplotypes of cultivated rice and 21 wild rice species, representing the whole SNPs region of *BADH1* by filtering miner allele frequency (maf) at 0.03. After filtering, there were in total 116 SNPs detected in the whole *BADH1* gene region. Condon changes together with amino acid transition due to functional SNP substitutions were also supported at each position of coding regions. Yellow regions indicated the same SNP compared wild rice, and green regions indicated specific regions compared to wild rice.

Supplementary Table S5B: List of haplogroups for the selected haplotypes of cultivated rice and 21 wild rice species, representing the whole InDel region of *BADH1* by filtering minor allele frequency (maf) at 0.03

Supplementary Table S6: Recorded mean values of eight different plant parameters under salt treatment (200 mM NaCl). During the measuring period, the samples were stored in incubators at 30 °C with 40% relative humidity for 10 days. Once the plumule emergence was 2 mm long, we started measuring the germination index (GI), and root length (RL) and shoot length (SL) were measured after 10 days. Total dry weight (TDW) was measured from RL and SL after drying at 80 °C for 24 h. All the remaining parameters, germination percentage (GP), germination energy (GE), mean germination time (MGT), and germination rate (GR) were measured and calculated after 10 days of salt treatment. The first letter "r" means "relative" because we converted our recorded mean values into "relative" values.

Supplementary Table S7: Pearson correlation coefficients among the tested plant parameters under the control (0 mM NaCl) and salt stress (200 mM NaCl) conditions. GP, germination percentage; GE, germination energy; GI, germination index; MGT, mean germination time; GR, germination rate; SL, shoot length; RL, root length; TDW, total dry weight.

Supplementary Table S8A: Association analysis between the recorded data of eight plant parameters and *BADH1* gene region. The associated positions of each parameter were selected at significant level by $p < 0.05$

Supplementary Table S8B: Summary of associated positions for all eight plant parameters. The number (0 or 1) indicates whether the respective position existed in association level or not at $p < 0.05$. There were five positions that have been published and confirmed for their association of this gene position with plant parameters under salt stress.

Supplementary Table S9A: The one-way ANOVA table for eight tested plant parameters based on the classified ecotypes

Supplementary Table 9B: The one-way ANOVA table for eight tested plant parameters based on the classified haplotypes.

Supplementary Figure S1: Gene structure of *BADH1* showing its published *BADH1* alleles that expressed significant association with salt tolerance in rice. Red boxes indicate 3′ and 5′ untranslated regions (UTR), blue boxes represent exons, and dark lines indicates intron regions. The arrowhead represents a negative DNA strand.

Supplementary Figure S2: Haplotype analysis of *BADH1 (Os04g0464200)* gene regions by 27 marker positions correlating to recorded plant major traits. Orange-colored positions indicate all SNP variations. Red-colored positions indicate deletions. Blue-colored positions indicate insertion. Blank cells are the same nucleotides to those mentioned in reference. "N" indicates the positions for "Unknown" nucleotides.

Supplementary Figure S3: Phylogenetic tree for the orthologous genes *BADH1* and BADH2. Scale bar indicates the proportion of sites changing along each branch. The relationship between *BADH1* and other *BADH1* homologues was inferred using ORTHOFINDER [1]. We used 19 species by searching the public databases, https://rice-genome-hub.southgreen.fr/node/70/53, accessed on 2 February 2021; http://rice.hzau.edu.cn; plants.ensembl.org and https://rapdb.dna.affrc.go.jp, accessed on 2 February 2021. It was a result of recent selection, as domestication of *BADH1* and *BADH2* has passed through selective pressures. In addition, *A. Thaliana AT1G74920.2* and *AT3G48170.1* have orthologs in *O. japonica*: *Os08t0424500-1* (*BADH2*), *Os04t0464200-01* (*BADH1*).

References

1. Emms, D.M.; Kelly, S. OrthoFinder: Phylogenetic orthology inference for comparative genomics. *Genome Biol.* **2019**, *20*, 1–14.
