# Supplementary material for: Haplotype Analysis of BADH1 by Next-Generation Sequencing Reveals Association with Salt Tolerance in Rice during Domestication
Source: Int J Mol Sci. 2021 Jul 15;22(14):7578. doi: 10.3390/ijms22147578 (PMC8305476; doi:10.3390/ijms22147578)
Supplement: Supplementary file 1 [file ijms-22-07578-s001.zip › Supplementary Table S7_Pearson correlation coefficients among the tested plant parameters.pdf]

Supplementary Table S7. Pearson correlation coefficients among the tested plant parameters under the control (0 mM NaCl) and salt stress (200 mM NaCl) conditions. GP, germination percentage; GE, germination energy; GI, germination index; MGT, mean germination time; GR, germination rate; SL, shoot length; RL, root length; TDW, total dry weight.

	Trait	GP	GE	GI	MGT	GR	SL	RL	TDW
Control	GP								
	GE	.946***							
	GI	.624***	.710***						
	MGT	-.294***	-.440***	-.926***					
	GR	.290***	.432***	.927***	-.999***				
	SL	-.001ns	.037ns	.168**	-.200***	.202***			
	RL	.073ns	.087ns	.110ns	-.100ns	.100ns	.254***		
	TDW	.305***	.265***	.088ns	.025ns	-.031ns	-.078ns	-.043ns	
200 mM	GP								
	GE	.394***							
	GI	.758***	.849***						
	MGT	-.577***	-.854***	-.943***					
	GR	.553***	.890***	.947***	-.996***				
	SL	.439***	.738***	.725***	-.718***	.737***			
	RL	.357***	.618***	.594***	-.576***	.598***	.712***		
	TDW	.785***	.235***	.586***	-.471***	.438***	.327***	.239***	
