# Supplementary figures and images for: Haplotype Analysis of BADH1 by Next-Generation Sequencing Reveals Association with Salt Tolerance in Rice during Domestication
Source: Int J Mol Sci. 2021 Jul 15;22(14):7578. doi: 10.3390/ijms22147578 (PMC8305476; doi:10.3390/ijms22147578)

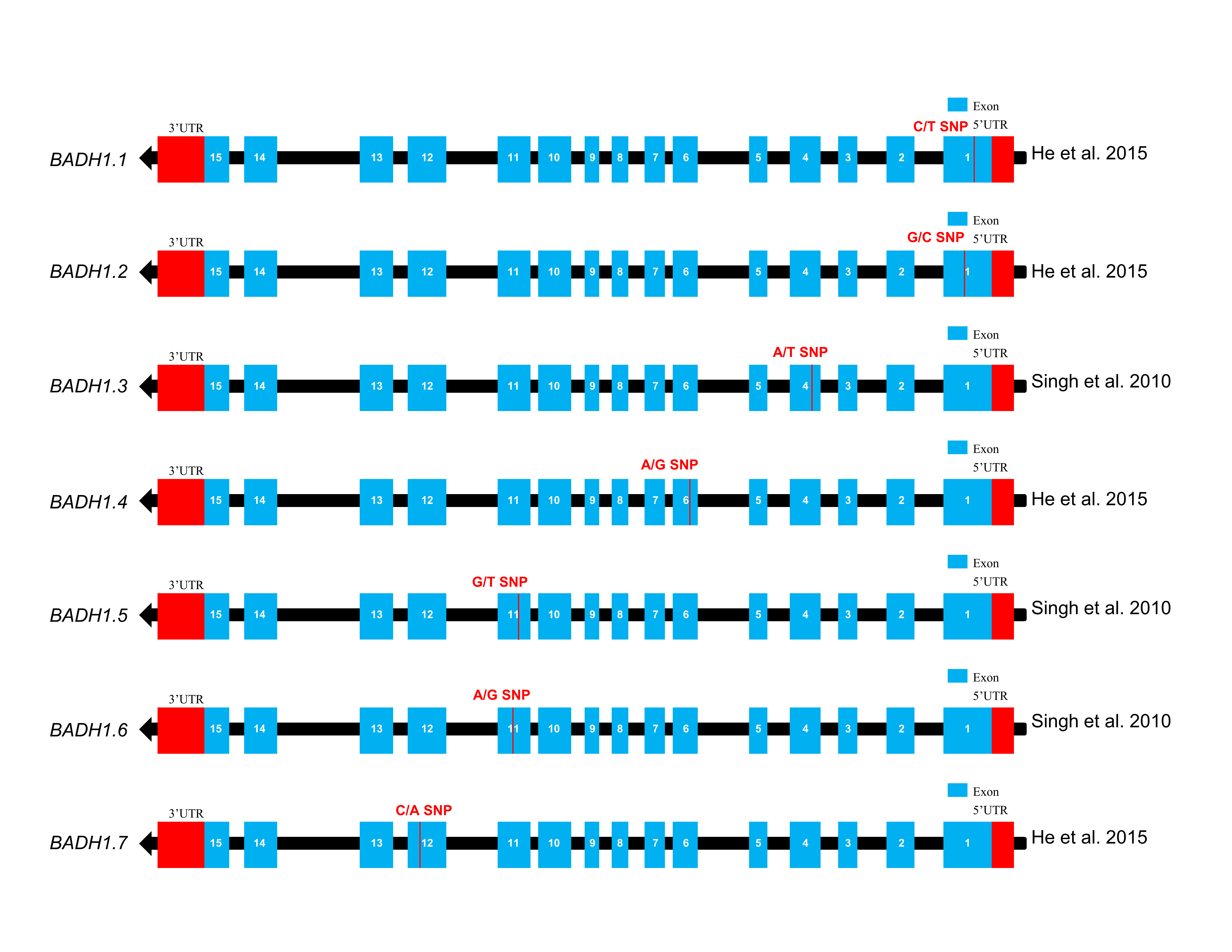

Supplement: Supplementary file 1 [file ijms-22-07578-s001.zip › Supplementary_Figure_S1.TIF]

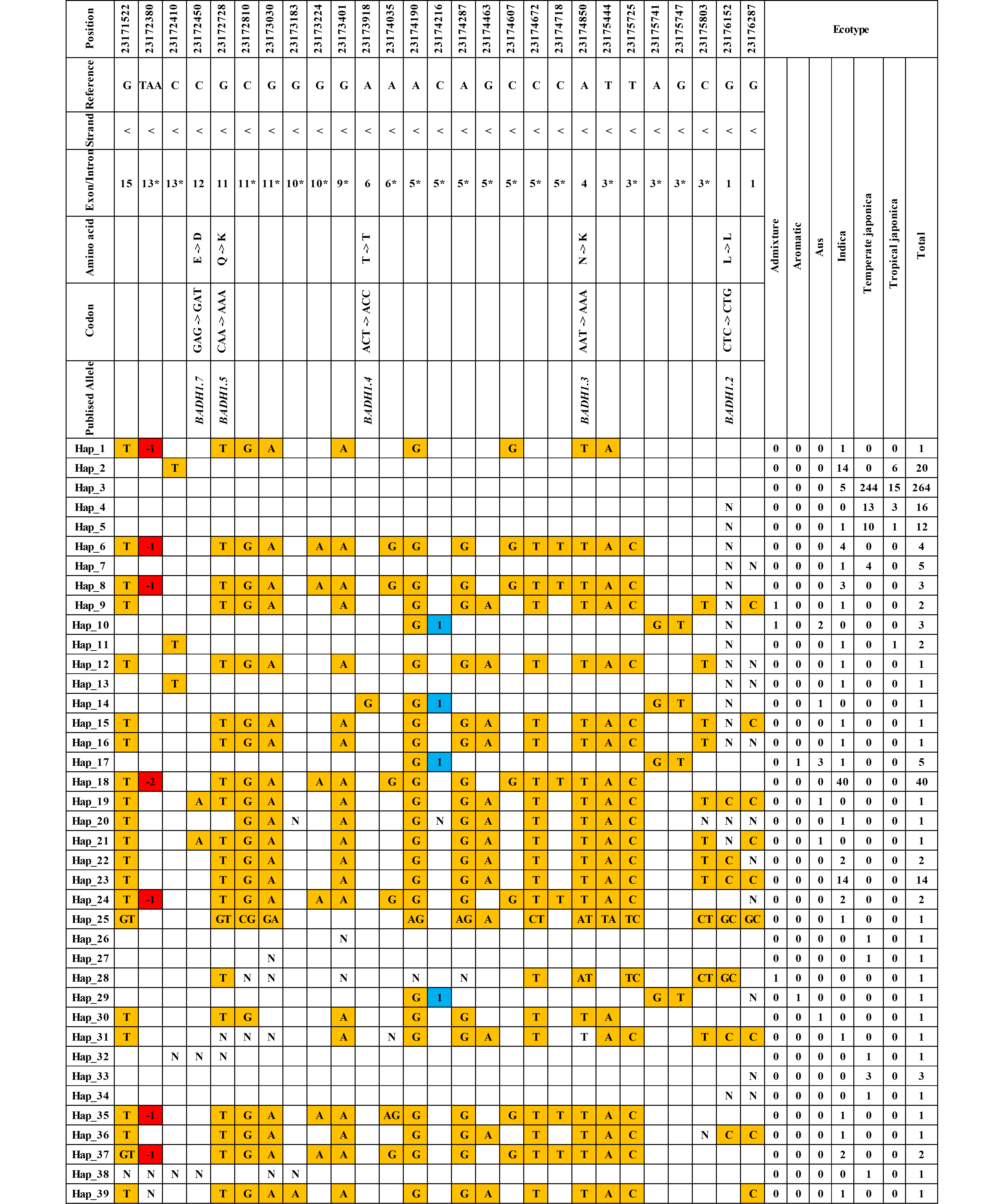

Supplement: Supplementary file 1 [file ijms-22-07578-s001.zip › Supplementary_Figure_S2.TIF]

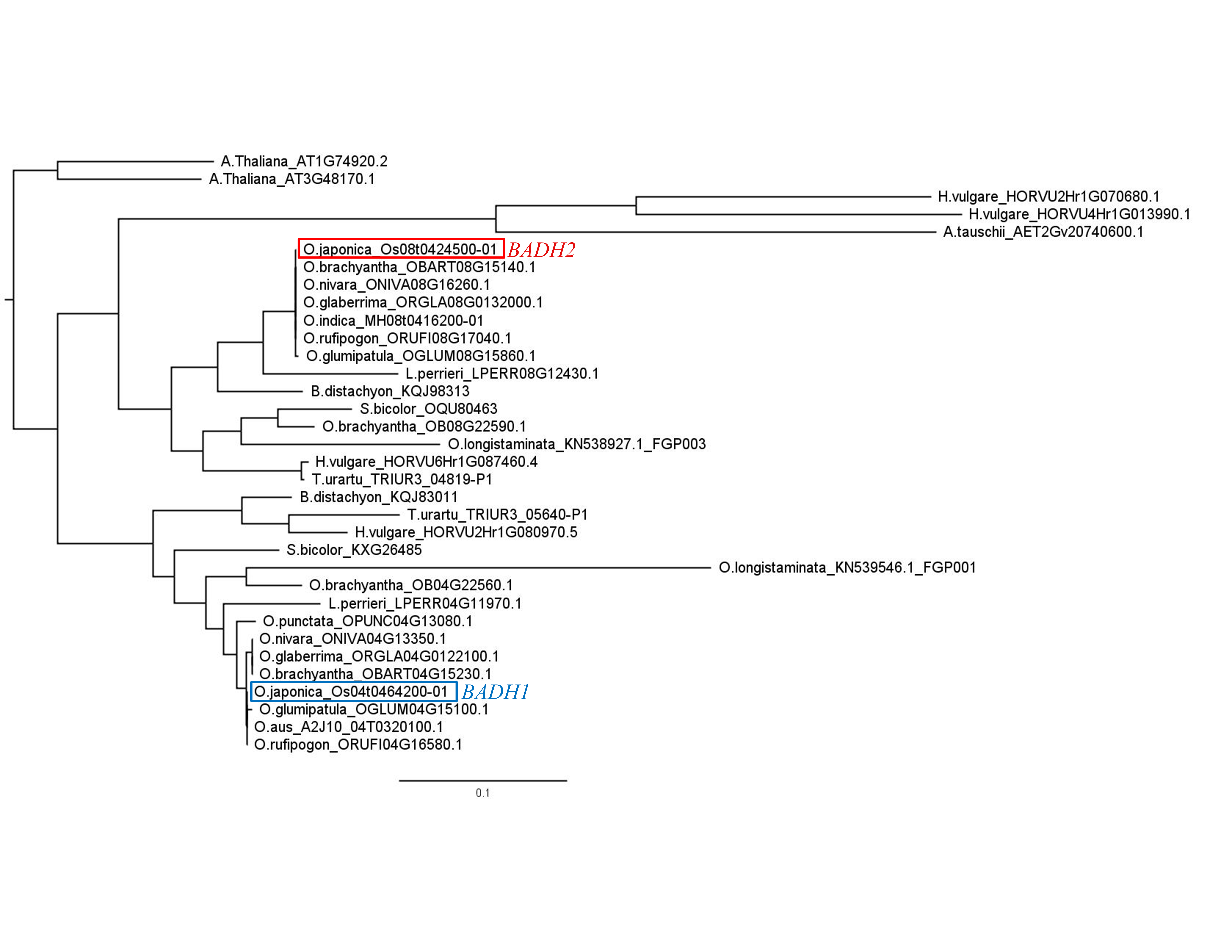

Supplement: Supplementary file 1 [file ijms-22-07578-s001.zip › Supplementary_Figure_S3.TIF]
